# Prolonged High-Fat Diet Consumption throughout Adulthood in Mice Induced Neurobehavioral Deterioration via Gut-Brain Axis

**DOI:** 10.3390/nu15020392

**Published:** 2023-01-12

**Authors:** Haicui Wu, Wenxiu Zhang, Mingyue Huang, Xueying Lin, Jiachi Chiou

**Affiliations:** 1 Shenzhen Key Lab for Food Biological Safety Control, Hong Kong Polytechnic University Shenzhen Research Institute, Shenzhen 518000, China; 2 Department of Food Science and Nutrition, The Hong Kong Polytechnic University, Hung Hom, Kowloon, Hong Kong, China; 3 Research Institute for Future Food, The Hong Kong Polytechnic University, Hung Hom, Kowloon, Hong Kong, China

**Keywords:** HFD, neurobehaviors, gut microbiota, gut-brain axis

## Abstract

Neuropsychiatric disorders have been one of the worldwide health problems contributing to profound social and economic consequences. It is reported that consumption of an excessive high-fat diet (HFD) in middle age could induce cognitive and emotional dysfunctions, whereas the mechanisms of the effects of long-term HFD intake on brain disorders have not been fully investigated. We propose a hypothesis that prolonged HFD intake throughout adulthood could lead to neurobehavioral deterioration via gut-brain axis. In this study, the adult C57BL/6J mice consuming long-term HFD (24 weeks) exhibited more anxiety-like, depression-like, and disruptive social behaviors and poorer performance in learning and memory than control mice fed with a normal diet (ND). In addition, the homeostasis of gut microbiota was impaired by long-term HFD consumption. Changes in some flora, such as *Prevotellaceae_NK3B31_group* and *Ruminococcus*, within the gut communities, were correlated to neurobehavioral alterations. Furthermore, the gut permeability was increased after prolonged HFD intake due to the decreased thickness of the mucus layer and reduced expression of tight junction proteins in the colon. The mRNA levels of genes related to synaptic-plasticity, neuronal development, microglia maturation, and activation in the hippocampus and prefrontal cortex of HFD-fed mice were lower than those in mice fed with ND. Interestingly, the transcripts of genes related to tight junction proteins, *ZO-1* and *Occludin* involved in blood-brain-barrier (BBB), were decreased in both hippocampus and prefrontal cortex after long-term HFD consumption. Those results indicated that chronic consumption of HFD in mice resulted in gut microbiota dysbiosis, which induced decreased expression of mucus and tight junction proteins in the colon, in turn leading to local and systemic inflammation. Those changes could further contribute to the impairment of brain functions and neurobehavioral alterations, including mood, sociability, learning and memory. In short, long-term HFD intake throughout adulthood could induce behavioral phenotypes related to neuropsychiatric disorders via gut-brain axis. The observations of this study provide potential intervention strategies to reduce the risk of HFD via targeting the gut or manipulating gut microbiota.

## 1. Introduction

It has been reported by The Lancet Commission that changing the factors of lifestyle, including diet, might prevent one-third of cases of global dementia [1]. A flood of research has revealed the correlations between excessive high-fat diet (HFD) and neuropsychiatric disorders associated with mood (e.g., depression and anxiety), development (e.g., autism spectrum disorder (ASD)) and neurodegeneration (e.g., Parkinson disease (PD) and Alzheimer disease (AD)) [2,3,4]. The detrimental neurobehavioral effects of HFD intake might occur in adults but not in aged mice [5]. A study implied that long-term HFD consumption (6 and 9 months) throughout adulthood impaired neurobehaviors, including greater depression- and anxiety-like behaviors and poorer memory performance [6]. However, the potential mechanisms involved in the effects of prolonged HFD intake on brain health are far from being completely elucidated.

HFD could disturb the balance of gut microbiota, thereby affecting neurobehaviors and brain functions via gut-brain axis. Transplantation of dysbiotic gut microbiota of obese mice to the mice with normal body weight results in cognitive impairment and impaired intestinal barrier [7]. In addition, the roles of the gut microbiota in the cross-link between Westernized dietary patterns, mood and cognitive function probably result from changes in levels of neuroactive metabolites, such as neurotransmitters and short-chain fatty acids, neuro-inflammation induced by the activation of a pro-inflammatory milieu, and overproduction of glucocorticoids in HPA-axis [8]. Previous studies in antibiotic-treated or germ-free mice have illustrated that gut microbiota play a crucial role in neuronal development and hippocampal neurogenesis via microglia activation [9,10]. The inflammation in the brain can also be influenced by gut microbiota through modulating microglia maturation and function [11]. Thus, HFD-induced gut microbiota dysbiosis is regarded as one of the major contributors to neurobehavioral alteration.

Gut microbiota dysbiosis and impaired intestinal barrier could induce leaky gut. Though the entry of exterior antigens from the gut lumen into the host could trigger both local and systemic inflammatory actions, the epithelial barrier and intestinal mucus serve as the gatekeeper to balance this immune response. Metabolic endotoxemia is a condition in which blood lipopolysaccharide (LPS) levels are elevated, resulting from increased gut permeability, consequently inducing neuroinflammatory responses [12]. In addition, the activation of microglia and astrocytes by systemic LPS has been reported to induce the expression of pro-inflammatory cytokines in the hippocampus of mice [13]. Valcarcel-Ares et al. also reported that increased expression of pro-inflammatory cytokines in obese mice is linked to synaptic impairment [14]. Altogether, systemic and neuroinflammatory responses and microglial/synaptic impairments can be stimulated through dysregulation of gut-brain axis with the potential to lead to neuropsychiatric disorders related to behavioral phenotypes in rodents.

In this study, we aim to assess the effects of long-term HFD consumption in C57BL/6J mice throughout adulthood on neurobehaviors via gut-brain axis and investigate the potential underlying mechanisms with a focus on the gut permeability, gut microbial-induced colonic and neuronal inflammation, and microglial/synaptic impairments.

## 2. Materials and Methods

### 2.1. Animals

Fourteen male C57BL/6J mice aged 7-week-old were purchased from Weitong Lihua Limited Company (Beijing, China). Three to four mice were housed per cage under the condition of sustained humidity (50 ± 15%), temperature (22 ± 2 °C), and light cycle (12:12 light/dark schedule) for 1 week. After acclimatization, mice were randomly divided into 2 groups, ND and HFD groups. Mice in ND and HFD groups were fed a control standard diet (10% kcal from fat, D12450J, Changzhou SYSE Bio-tec) or high-fat diet (60% kcal from fat, PD6001, Changzhou SYSE Bio-tec), respectively. The duration of dietary treatment was from young adulthood (2-month-old) to maturity (6-month-old). In the last two weeks, all mice underwent behavioral tests. Subsequently, blood samples were drawn by cardiac puncture, and tissues were collected after they were sacrificed with carbon dioxide. Experimental procedures used in this study were approved by the Animal Subjects Ethics Committee of The Hong Kong Polytechnic University (ASESC No. 21-22/74-ABCT-R-OTHERS).

### 2.2. Behavioral Measurements

#### 2.2.1. Open Field Test (OFT)

The OFT measures locomotor activity to evaluate anxiety- or depression-like behavior [15]. The mouse was carefully put into the center of a plastic gray box of 40 cm × 40 cm × 40 cm to explore freely for 5 min. This arena was divided into two zones, a center zone, and a peripheral zone. The total distance, speed, time and distance spent in the center were analyzed with a video-imaging system (EthoVisionXT; Noldus Information Technology, Wageningen, The Netherlands).

#### 2.2.2. Elevated Plus Maze (EPM) Test

The EPM test is used to measure anxiety-like behavior in rodents [15]. The EPM elevated 50 cm from the floor, consisted of two open and closed arms; the size of each arm is 40 cm × 10 cm × 25 cm (L × W × H) in closed arms. The mouse was placed in the central area of the maze facing one of the open arms and exploring freely for 5 min. The total distance and the distance, time, and entry times in open arms were recorded with EthoVisionXT video-imaging system.

#### 2.2.3. Three-Chamber Social Test

The three-chamber social test was developed for social interaction and social novelty preference of rodents [16]. The mouse was gently put into the empty arena, which was a 60 × 40 cm^2^ plastic box divided into three equal chambers, to adapt for 5 min. After 5 min, the mouse could interact with either an empty wire cup or the other cup with a stranger. The time spent on the empty cup or the stranger was recorded with a video-imaging system. In the last 5 min, a second stranger was introduced in the empty cup, and the time the subject spent on stranger 1 and stranger 2 was recorded using EthoVisionXT video-imaging system. Sociability index (%): [(time spent with mouse) − (time spent with empty cup)]/[(time spent with mouse) + (time spent with empty cup)] ∗ 100 or [(time spent with novel mouse) − (time spent with familiar mouse)]/[(time spent with novel mouse) + (time spent with familiar mouse)] ∗ 100.

#### 2.2.4. Nesting Test and Marble Burying

Nests are important for mice to conserve heat, reproduce, and take shelter. Nesting representing normal social behavior could be measured easily in the home cages of mice, particularly with the advent of pressed cotton materials. The mice were housed individually, and the cotton materials were placed inside the cage. After 24 h, the mice were transferred to a new cage, and pictures of those materials were taken and scored [17].

Marble burying represents the repetitive behaviors that could be observed in obsessive-compulsive disorder (OCD) and autism spectrum disorders (ASD) [18]. Mice were put into a single cage with 10 glass beads on the bedding. The number of beads buried in a 30 min session is scored.

#### 2.2.5. Novel Object Recognition (NOR) Test

The NOR tests long-term learning and memory in mice [19]. This test requires 3 continuous days, namely habituation day, training day, and testing day. On the habituation day, the mouse was put in the arena (the size is the same as the arena in OFT) gently for 5 min to freely explore. On the second training day, the mouse was put into an arena with two identical objects placed in central symmetrical positions. It takes the mouse 5 min to explore freely in this arena. On the last testing day, one of the objects was changed to a novel object, and the mouse was also allowed to explore freely for 5 min. The motility of mouse was recorded using EthoVisionXT video-imaging system. Discrimination ratio was calculated by the following: [(the time spent exploring the novel object) − (the time spent exploring the familiar object)]/(the total exploring time).

#### 2.2.6. Morris Water Maze (MWM)

It is widely used to study memory and spatial learning through the MWM [20]. The circular tank is 120 cm in diameter and 50 cm in depth, filled with water. Food-grade titanium dioxide was added to water to make it opaque. The maze was divided into four quadrants. A platform of 4.5 cm in diameter and 14.5 cm in height was placed in the quadrant. This test was conducted for 6 days. On the first day, the platform was put on top of water, and the water was undyed. All mice could see the platform and stand on it for 60 s to accommodate the environment. In the following 4 days, the platform was invisible, and the water was white-dyed. The mouse was placed into an assigned quadrant, swim freely for 60 s to find the platform. On the last day, the platform was removed, and the mouse was put into the tank for 60 s. The time and distance spent in the target quadrant were recorded with EthoVisionXT video-imaging system.

### 2.3. Reverse Transcription Quantitative Polymerase Chain Reaction (RT-qPCR)

Colon and brain tissues were homogenized with beads in Trizol (Thermo Fisher Scientific, Waltham, MA, USA). Total RNA was extracted, and concentration was measured. cDNA was obtained from one microgram of RNA using cDNA Reverse Transcription Kit (Takara PrimeScript RT Master Mix, Dalian, China). The mRNA expression was quantified by RT-qPCR using the SYBR green PCR kit (Biotium Forget-Me-Not™ EvaGreen^®^ qPCR Master Mix, Fremont, CA, USA) in a real-time PCR detection system (Bio-Rad, Hercules, CA, USA). GAPDH was used as the internal control, and the relative mRNA expression level was determined with the 2^−ΔΔCt^ method.

### 2.4. ELISA Assay

LPS was determined in plasma by enzyme-linked immunosorbent assay (Limulusassay kit, Cat. 18110115, China) according to the manufacturer’s instructions. The levels of TNF-α, IL-6, and IL-1β in plasma were determined using ELISA kits (Jiangsu Meimian Industrial Co., Ltd., Yancheng, China).

### 2.5. Hematoxylin and Eosin (H&E) and Alcian Blue (AB) Staining

Epididymal fat, liver and colon were fixed in 10% formalin and embedded in paraffin, followed by sectioned into 5 μm, hydrated with xylene and gradient concentrations of ethanol. H&E was used for histopathological staining. ImageJ was used to evaluate epididymal adipocyte size and the length of villi in colon.

To measure the thickness of colonic mucus layer, the sectioned colonic samples were deparaffinized and hydrated with distilled water. They were further placed in 3% acetic acid solution for 5 min, followed by staining in Alcian blue solution. The samples were rinsed in distilled water and counterstained with filtered nuclear fast red solution. The thickness of the colonic sections was measured using ImageJ.

### 2.6. Immunohistochemical Staining

Frozen brain tissue was cut into 20 μm sections using a cryostat. Sodium Citrate Antigen Retrieval Solution (PH 6.0) was performed to retrieve antigens and quench the brain samples with 3% peroxide-methanol. After washing with PBS, the sections were incubated with primary antibody (anti-Iba1, 1:500, Servicebio, GB11105) at 4 °C overnight. The corresponding secondary antibody was applied and co-incubated with the samples at RT for 30 min. The sections were colored with DAB kit (Servicebio, Wuhan, China). The images were observed under a light microscope.

### 2.7. 16SrRNA Sequencing Analysis

Samples were collected from rectum before sacrifice. Genomic DNA was extracted using the TIANamp Stool DNA kit (Tiangen, Beijing, China) according to manufacturer’s instructions. The DNA extract was checked on 1% agarose gel, and DNA concentration and purity were determined with NanoDrop 2000 UV-vis spectrophotometer (Thermo Scientific, Wilmington, DE, USA). The hypervariable region V3-V4 of the bacterial 16S rRNA gene was amplified with primer pairs 338F (5′-ACTCCTACGGGAGGCAGCAG-3′) and 806R (5′-GGACTACHVGGGTWTCTAAT-3′) at Majorbio BioPharm Tech. Co. (Shanghai, China). Purified amplicons were pooled in equimolar and paired-end sequenced on an Illumina MiSeq PE300 platform/NovaSeq PE250 platform (Illumina, San Diego, CA, USA) according to the standard protocols by Majorbio Bio-Pharm Technology Co. Ltd. (Shanghai, China). The raw 16S rRNA gene sequencing reads were demultiplexed, quality-filtered by fastp version 0.20.0 [21], and merged by FLASH version 1.2.7 [22] with the following criteria: (i) the 300 bp reads were truncated at any site receiving an average quality score of <20 over a 50 bp sliding window, and the truncated reads shorter than 50 bp were discarded, reads containing ambiguous characters were also discarded; (ii) only overlapping sequences longer than 10 bp were assembled according to their overlapped sequence. The maximum mismatch ratio of overlap region is 0.2. Reads that could not be assembled were discarded; (iii) Samples were distinguished according to the barcode and primers, and the sequence direction was adjusted, exact barcode matching, 2 nucleotide mismatch in primer matching. Operational taxonomic units (OTUs) with 97% similarity cutoff [23] were clustered using UPARSE version 7.1 [23], and chimeric sequences were identified and removed. The taxonomy of each OTU representative sequence was analyzed by RDP Classifier version 2.2 [24] against the 16S rRNA database using confidence threshold of 0.7.

### 2.8. Data Analysis

All values represent the means and SEM. Data were compared by a two-tailed unpaired Student’s *t*-test as appropriate (*p* < 0.05 was deemed significant) using GraphPad Prism 5 (GraphPad Software). When there is more than one factor, the level of statistical significance between groups was determined using one-way or two-way analysis of variance (ANOVA) followed by a post hoc Tukey test. Correlations between parameters were assessed using Pearson’s correlation test. Statistical analysis for gut microbiome was conducted with STAMP, and functional differences in orthologs among groups were assessed by a one-way ANOVA followed by post hoc Tukey–Kramer or Kruskal–Wallis test for multiple comparisons.

## 3. Results

### 3.1. Prolonged HFD Intake Induces Metabolic Alterations

**Figure 1:**
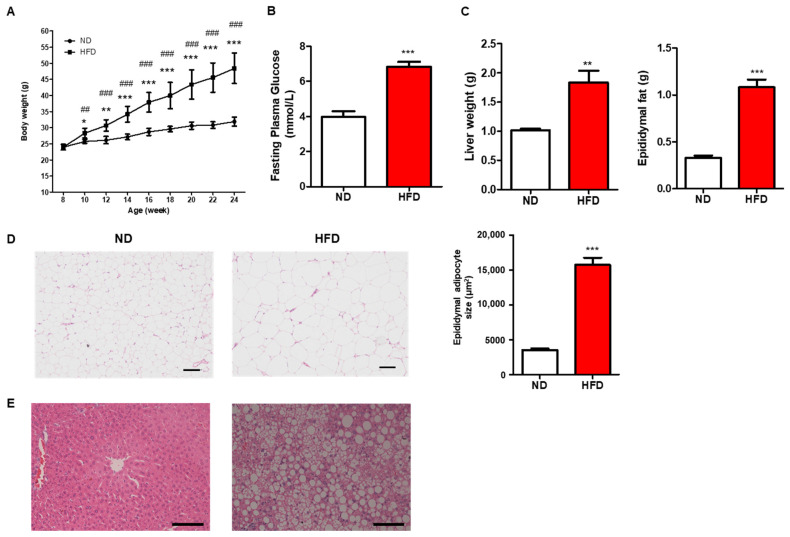
Effect of long-term HFD consumption on metabolic parameters. Body weight (**A**), fasting blood glucose (**B**), liver weight and white fat weight (**C**), adipocyte size of white fat (**D**), and hepatic steatosis (**E**). Values are shown as the mean ± SEM. One-way ANOVA with a post hoc Tukey test or *t*-test was performed. * *p* < 0.05; ** *p* < 0.01; *** *p* < 0.001 vs. ND, ## *p* < 0.01; ### *p* < 0.001 vs. age 8. Bar: 100 μm.

All mice were weighed biweekly from eight-week-old to the end of the experiments. The body weight of mice in both ND and HFD groups did not show any difference at the beginning of the experiment, while long-term HFD consumption induced significantly increased body weight from the second week of treatment (Figure 1A). Also, the fasting glucose and tissue weight, including liver and epididymal fat, were significantly higher in the HFD group than in ND (Figure 1B,C). Consistent with the changes in epididymal fat and liver, increased adipocyte size and aggravated fatty liver by H&E staining were observed (Figure 1D,E). Those results indicate that prolonged HFD intake induced metabolic disorders.

### 3.2. Prolonged HFD Intake Induces Neurobehavioral Disorders

#### 3.2.1. Anxiety- and Depression-like Behavior

**Figure 2:**
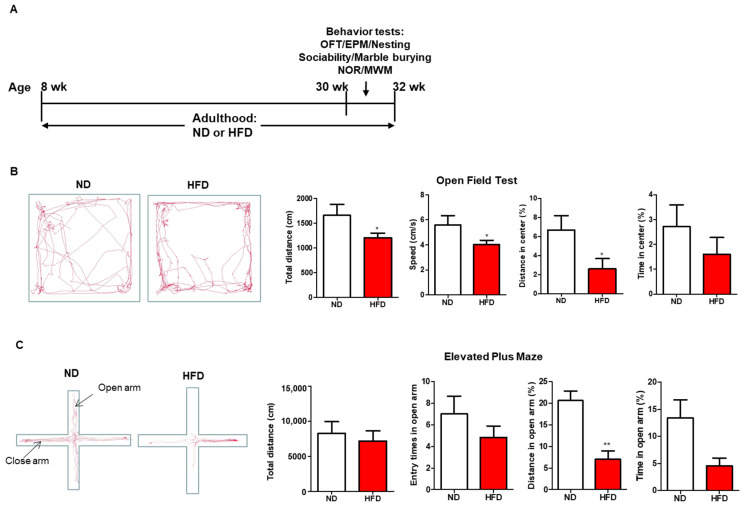
Effect of long-term HFD consumption on anxiety- and depression-like behavior in mice. Experimental design (**A**). Representative movement trace images, total distance, speed, percentage of distance in center, and time in center in Open Field Test of ND- and HFD-fed mice (**B**). Representative movement trace images, total distance, entry times in open arm, percentage of distance in open arm, and time in open arm of Elevated Plus Maze test (**C**). ND, normal diet; HFD, high-fed diet. Values are shown as the mean ± SEM. *t*-test was performed. * *p* < 0.05; ** *p* < 0.01.

The timeline of this study is shown in Figure 2A. The mice were treated with HFD or ND from 2-month-old to 8-month-old, which covered their entire adulthood. Behavioral tests were conducted in the last two weeks before sacrifice. OFT and EPM are two common approaches to evaluate anxiety- and depression-like behavior in rodents. The mice showing anxiety- or depression-like behavior spent more time in the peripheral zones of OFT or hid in closed arms of EPM than normal mice. Mice of HFD consumption showed a reduced total distance and speed than those fed with ND in OFT significantly (Figure 2B). In addition, the distance and time spent in central zones were significantly decreased in HFD mice in comparison to those in the ND group (Figure 2B). In EPM, HFD-fed mice spent notably less distance in the open arm than those fed with ND, although the fewer entry times and time spent in the open arm of HFD mice were not statistically different from those in ND mice (Figure 2C). These results suggested that long-term HFD intake could lead to anxiety- and depression-like behavior in mice.

#### 3.2.2. Autism-like or Social Behavior

**Figure 3:**
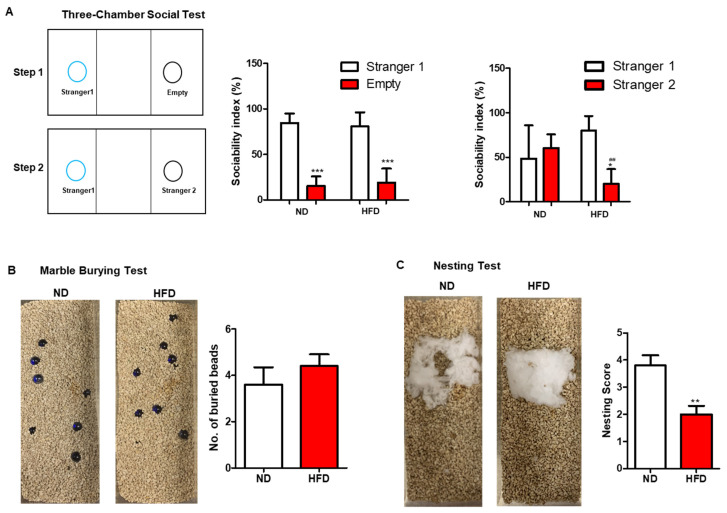
Effect of long-term HFD consumption on autism-like behavior in mice. Schematic diagram, sociability index between stranger 1 and empty cage, and sociability index between stranger 1 and stranger 2 of three-chamber social test (**A**). Representative images and number of burying beads in marble burying test (**B**). Representative images and nesting score in nesting test of ND- and HFD-fed mice (**C**). Values are shown as the mean ± SEM. *t*-test or two-way ANOVA with a post hoc Tukey test was performed. * *p* < 0.05; ** *p* < 0.01; *** *p* < 0.001 vs. ND, ## *p* < 0.01 vs. stranger 1.

Mice with autism-like behavior would display poorer social skills or compulsive-like activity. In the three-chamber social test, both ND- and HFD-fed mice exhibited good sociability between an empty cup and a cup with a stranger mouse. However, they showed lower sociability for social novelty between a familiar mouse and a stranger mouse in HFD-fed mice though the sociability index for a stranger mouse was slightly increased in ND-fed mice insignificantly (Figure 3A). Prolonged HFD consumption increased the number of buried beads in marble burying test compared to those fed with ND, though the difference was not significant (Figure 3B). Moreover, the normal nesting behavior was also impaired in mice of the HFD group significantly (Figure 3C). These results showed that mice with long-term HFD consumption exhibited impaired social behavior in nesting test but not in three-chamber social test. On the other hand, the compulsive-like activity measured by marble burying test did not change significantly in HFD-fed mice when compared with those in the ND group. Altogether, long-term HFD consumption might induce autism-like behavior.

#### 3.2.3. Learning and Memory

**Figure 4:**
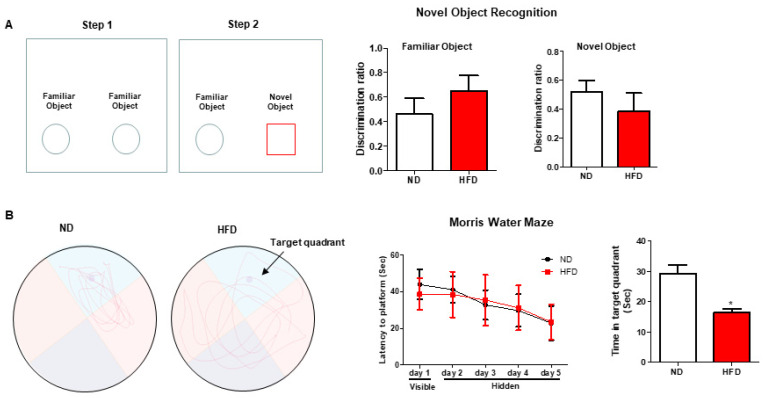
Effect of long-term HFD consumption on learning and memory in mice. Schematic diagram, discrimination ratio between familiar objects, and discrimination ratio between familiar object and novel object in Novel Object Recognition (**A**). Representative movement trace images, latency to platform from day 1 to day 6, and time spent in target quadrant on day 6 in Morris Water Maze (**B**). Values are shown as the mean ± SEM. *t*-test or one-way ANOVA with a post hoc Tukey test was performed. * *p* < 0.05 vs. ND.

The working and spatial memory were assessed by the NOR and MWM tests, respectively. Compared with the ND-fed mice, the mice fed with long-term HFD exhibited a slightly decreased discrimination index between a familiar object and a novel object in NOR test (Figure 4A). In MWM, the mice in both ND and HFD groups exhibited good performance in learning since the latency to platform decreased from day 1 to day 5 in those mice (Figure 4B). However, on the last day, mice fed with prolonged HFD spent less time in the target quadrant than those fed with ND, suggesting that HFD impaired the ability of memory (Figure 4B). Thus, those results indicated that HFD might affect the function of memory.

### 3.3. Prolonged HFD Diet Induces Gut Microbiota Dysbiosis

**Figure 5:**
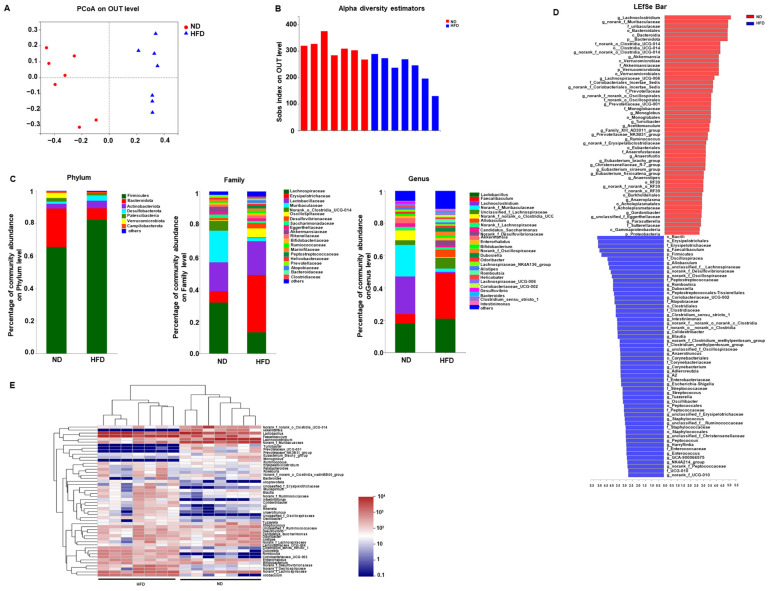
Effect of long-term HFD consumption on gut microbiota in mice. Principal coordinates analysis plot of unweighted UniFrac distances (**A**), Alpha diversity in ND- and HFD- fed mice using Sobs index on OUT level (**B**), Community alterations at Phylum, Family, and Genus level (**C**), Linear discriminant analysis (LDA) effect size showing the most differentially significant abundant taxa (**D**), Community heatmap analysis on Genus level in mice (**E**).

Bacterial 16S rRNA Sequencing was performed to investigate the effects of HFD on gut microbiota. A clear separation between the gut microbiota of ND-fed mice and HFD-fed mice was observed on the Bray–Curtis distance, suggesting a compositional difference in the β-diversity in these two groups (Figure 5A). The α-diversity assessed by the Sobs index was decreased in HFD-fed mice compared with that in ND-fed mice, implying the HFD may reduce the biodiversity of gut microflora (Figure 5B). The community changes of gut microbiota were analyzed from Phylum to Genus level. At the phylum level, we observed that HFD consumption increased the relative abundance of Firmicutes but decreased that of Bacteroidota and Verrucomicrobiota (Figure 5C). At the family level, the abundance of Lachnospiraceae, Muribaculaceae, and Akkermansiaceae was decreased in mice with long-term HFD intake. On the other hand, HFD consumption increased the relative abundance of Family Erysipelotrichaceae and Oscillospiraceae (Figure 5C). Specifically, results at Genus levels showed that HFD increased the relative abundance of *Faecalibaculum*, *unclassified_f_Lachnospiraceae*, and *Allobaculum* while the abundance of *Lachnoclostridium*, *norank_f_Muribaculaceae*, *Akkermansia* in the gut microbiota of mice fed with HFD decreased compared with those fed with ND. In addition, when comparing the microbiota of ND- and HFD-fed mice using an LDA effect size (LEfSe) calculation, we found 107 differentially abundant taxonomic classes with an LDA score higher than 2.0, among which 51 taxonomic classes were significantly abundant in the ND group while 56 classes were significantly abundant in HFD group (Figure 5D). The community heatmap analysis showed that a remarkable difference in the gut bacterial community between HFD-fed and ND-fed mice at the genus level was found (Figure 5E). For example, *Akkermansia*, *Turicibacter*, *Prevotellaceae_UCG-001*, *Prevotellaceae_NK3B31_group*, and *Ruminococcus* exhibited high abundance in ND-fed mice, but low abundance in HFD-fed mice, while *unclassified_f_Erysipelotrichaceae*, *Blautia*, *A2*, *Tuzzerella*, *Rombousia* showed high abundance in HFD treated mice but not in control mice (Figure 5E). Taken together, those results showed that prolonged HFD could induce dysbiotic microbiota in the gut.

### 3.4. Correlation Analysis between the Gut Microbiota Composition and Neurobehaviors

**Figure 6:**
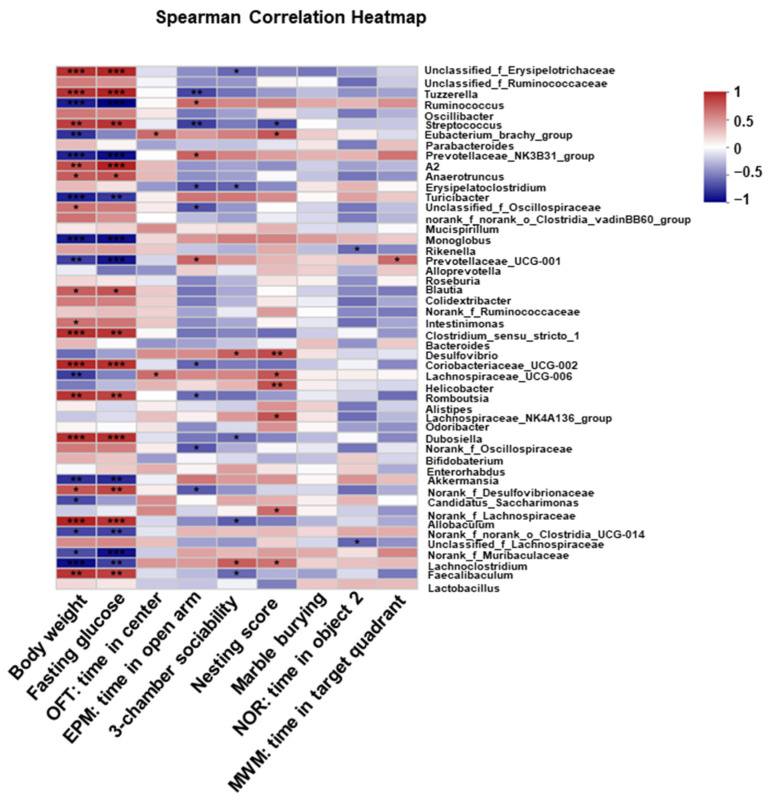
Effect of long-term HFD consumption on functions of gut microbiota in mice. Spearman correlation heatmap showing the correlations between neurobehavioral alterations and specific gut flora. * *p* < 0.05; ** *p* < 0.01; *** *p* < 0.001.

Following our observation that HFD induced structural and functional changes in gut microbiota, we examined the correlations between gut microbiota and body weight, fasting glucose, and neurobehaviors. Overall, a high correlation between gut bacterial communities and body weight or fasting glucose was found, as many taxa at the genus level were significantly correlated to these two parameters. However, a statistical correlation between the taxa and marble burying was observed, suggesting a low association between gut microbiota and this neurobehavior (Figure 6). Notably, the abundance of *Prevotellaceae_NK3B31_group*, *Ruminococcus*, and *Prevotellaceae_UCG-001* were negatively correlated with increased body weight and high level of fasting glucose but positively correlated with time spent in the open arm of EPM. *Prevotellaceae_UCG-001* were positively associated with time spent in the quadrant in MWM. In addition, the correlation between body weight or fasting glucose and the Genus *Lachnoclostridium* was negative, whereas a positive correlation between this genus and sociability in three chamber social tests and nesting scores in nesting was observed (Figure 6). Overall, these results suggested that there are potential functional interactions between gut microbiota composition and host physiology which could affect neurobehavior.

### 3.5. Prolonged HFD Intake Impairs Gut Barrier Integrity and Induces Inflammatory Responses in Mice

**Figure 7:**
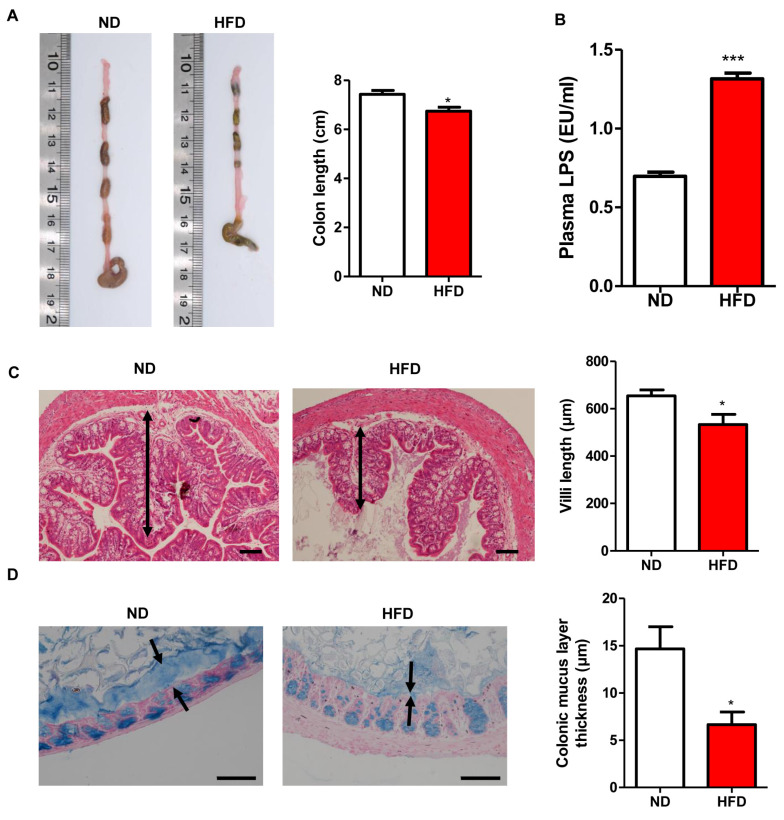
Effect of long-term HFD consumption on gut barrier integrity damage in mice. Colon length in ND and HFD fed mice (**A**), Plasma endotoxin level (**B**), villi length in proximal colon ((**C**), Arrows indicate the length of villi), thickness of mucus layer ((**D**), opposing black arrows with shafts delineate the mucus layer that was measured). Values are shown as the mean ± SEM. *t*-test was performed. * *p* < 0.05; *** *p* < 0.001. Bar: 100 μm.

**Figure 8:**
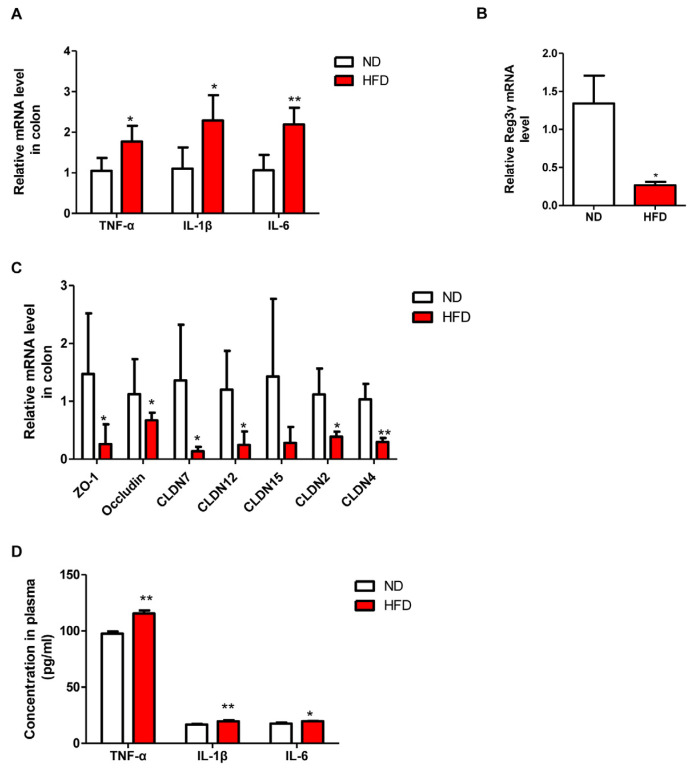
Effect of long-term HFD consumption on the expression of markers involved in inflammation and tight junction in colon. The mRNA levels of inflammation-related genes (**A**), the mRNA level of Reg3γ (**B**), the mRNA levels of tight junction-related genes (**C**). Levels of inflammatory cytokines in plasma (**D**). CLDN, claudin. Values are shown as the mean ± SEM. *t*-test was performed. * *p* < 0.05; ** *p* < 0.01.

Due to the high content of fat and low content of fiber in HFD, mice consuming long-term HFD exhibited significantly decreased length of colon compared with that in ND-fed mice (Figure 7A). Plasma endotoxin level was notably increased in HFD mice in comparison to that in control mice (Figure 7B). Furthermore, the length of villi and the thickness of the mucus layer in the proximal colon were significantly reduced in HFD mice than in ND-fed mice. Interestingly, mRNA levels of the genes related to inflammation (*TNF-α*, *IL-1β*, and *IL-6*) were significantly higher in HFD mice than those in the ND group (Figure 8A). This observation was further verified by determining the protein levels of TNF-α, IL-6, and IL-1β in the plasma, which showed that their expressions were significantly increased in the HFD group than those in ND-fed mice. On the other hand, mRNA levels of antimicrobial peptide *Reg3γ* and the genes involved in tight junction proteins, including *ZO-1*, *occludin*, *CLDN7*, *CLDN12*, *CLDN15*, *CLDN2*, and *CLDN4*, were decreased in HFD mice (Figure 8B,C). Altogether, these results demonstrated that prolonged HFD intake could impair gut barrier integrity and induce inflammatory responses in the colon.

### 3.6. Prolonged HFD Intake Affects the Function of Neurons and the Expression of Tight Junction Proteins Involved in Blood-Brain-Barrier

**Figure 9:**
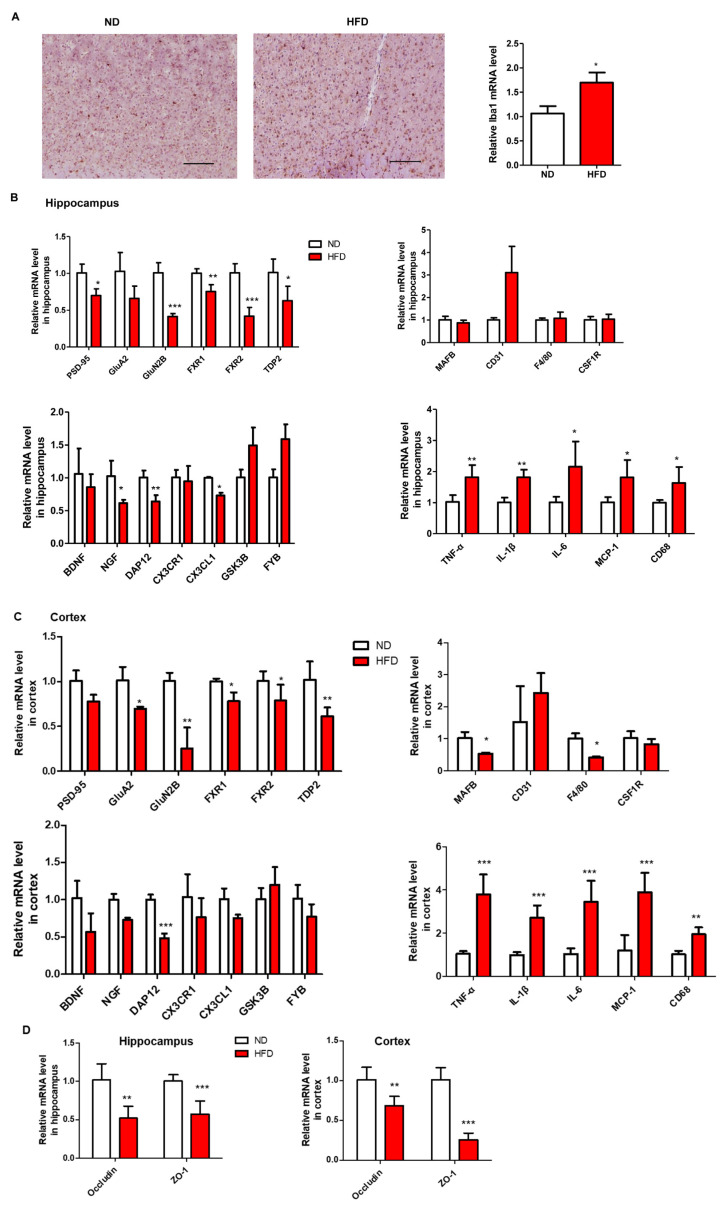
Effect of long-term HFD consumption on the expression of markers involved in microglia function and synapse damage in hippocampus and prefrontal cortex. Representative images of IHC of Iba-1 protein in prefrontal cortex and relative mRNA level of Iba-1 (**A**). The mRNA levels of synapse-, microglia maturation-, microglia function- and inflammation- related genes in the hippocampus (**B**) and in the prefrontal cortex (**C**). The mRNA levels of tight junction proteins, ZO-1 and Occludin, in hippocampus and prefrontal cortex (**D**). Values are shown as the mean ± SEM. *t*-test was performed. * *p* < 0.05; ** *p* < 0.01; *** *p* < 0.001. Bar: 100 μm.

In this study, the protein level and relative mRNA expression of ionized calcium binding adaptor molecule 1 (*Iba-1*), a marker of activated microglia, was measured using IHC and RT-PCR, respectively [25]. The results showed that long-term HFD consumption could significantly induce the expression of Iba-1 in the cortex (Figure 9A). The mRNA levels of genes related to synaptic-plasticity (*PSD-95* and *FXR1*), neuronal development (*FXR2* and *TDP2*), NMDA receptor *GluN2B*, and AMPA receptor *GluA2* in the hippocampus and prefrontal cortex of HFD-fed mice were lower than those in mice fed with ND (Figure 9B,C). Moreover, the mRNA levels of the genes involved in microglia maturation (*MAFB*, *CD31*, *F4/80*, and *CSF1R*), microglia-neuron interaction (*BDNF*, *NGF*, *DAP12*, *CX3CR1*, and CX3CL1), microglia activation (*GSK3B* and *FYB*), neuroinflammatory cytokines (*TNF-α*, *IL-1β*, *IL-6*, *MCP-1*, and *CD-68*) were also evaluated in hippocampus and prefrontal cortex. Prolonged HFD treatment lowered the levels of *BDNF*, *NGF*, *DAP12*, and *CX3CL1* but increased *CD31*, *TNF-α*, *IL-1β*, *IL-6*, *MCP-1*, and *CD-68* in both hippocampus and prefrontal cortex (Figure 9B,C). Apart from the changes in microglia and synaptic markers, the mRNA levels of tight junction proteins involved in BBB were also evaluated. The results revealed that the expression levels of *ZO-1* and *occludin* in the hippocampus and prefrontal cortex were attenuated in mice fed with HFD for a long time than in mice fed with ND (Figure 9D). These findings indicated that long-term HFD could result in synaptic impairment, disruption of microglia function, and decreased expression of tight junction proteins in the hippocampus and prefrontal cortex.

## 4. Discussion

The results of this study showed that long-term HFD consumption in adult mice induced behavioral phenotypes related to neuropsychiatric disorders, such as anxiety- and depression-like behaviors, social behaviors, and impairment in learning and memory. In consistency with those neurobehavioral alterations, the genes involved in BBB, neuroinflammation, synaptic-plasticity, and microglia activation and maturation are also expressed differently in HFD and ND groups. Accumulative evidence suggests that brain disorders can be induced by gut microbiota dysbiosis and leaky gut. Our results further demonstrated the adverse effects of prolonged HFD consumption on structural and functional changes of gut microbiota, mucus thickness, and tight junction proteins in the colon. Furthermore, systemic inflammation was also observed in the present study, which might be induced by leaky gut. In short, we conclude that long-term consumption of HFD in mice throughout adulthood could impair neurobehaviors and brain functions via gut-brain axis.

It has been known that HFD consumption increases the risks of obesity, diabetes, and metabolic syndrome [26]. It is popular to choose a diet consisting high ratio of fat in mature adults in modern life [27]. Caloric intake increases beyond energy expenditure in adulthood, mainly due to time constraints, convenience, and modern lifestyles [28]. Further evidence indicates that diets rich in high fat have a profound impact on a number of psychiatric disorders, such as Alzheimer’s disease and other forms of cognitive impairment [29,30,31]. Given an emerging crisis of brain disorders and the increasing global burden of obesity induced by HFD intake, it is of utmost importance to elucidate the underlying mechanisms of HFD consumption on neurobehavioral alterations. Middle age is a crucial period that plays an important role in health later in the lifespan [32,33].

Consistent with previous studies, we found that HFD induces various neuropsychiatric behavioral abnormalities throughout the whole period of adulthood. OFT and EPM are two commonly-used approaches to assess depression- and anxiety-like behaviors in rodents [34]. In our study, long-term HFD consumption reduced the distance and time in the center of OFT and the open arm of EPM. However, it was noticed that the total distance and speed in OFT were significantly decreased in HFD-fed mice, which probably resulted from the decline of physical activity levels in overweight mice [35]. Moreover, the observations of mice in the three-chamber social test, marble burying and nesting test showed that mice consuming long periods of HFD exhibited less sociability to a stranger mouse than that in ND-fed mice, suggesting prolonged consumption of HFD leads to impaired social behaviors. The autism-like behaviors were evaluated by a marble burying test representing obsessive-compulsive disorder in the brain [18]. Interestingly, the number of buried beads in the HFD group was slightly more than in the ND group. Though accumulating epidemiological evidence suggests a possible link between being overweight and ASD, most of them illustrate the effect of maternal obesity on the autism-like behavior of offspring [36,37,38]. Thus, the consumption of HFD as a single factor in adult rodents might be insufficient to induce autism-like behavior. NOR and MWM are two common behavioral assays used for the investigation of various aspects of learning and memory in rodents [19,39]. In this study, the learning deficit was not observed in HFD-fed mice in MWM, whereas long-term consumption of HFD did impair the ability of memory as evaluated in both NOR and MWM.

The hippocampus and prefrontal cortex are two critical regions related to cognition and emotion in the brain, playing a central role in the etiology and pathophysiology of cognitive and emotional impairment [40,41]. Furthermore, despite a number of studies demonstrating that HFD-related neurobehavioral changes are associated with neural plasticity in the hippocampus and prefrontal cortex [40,42], the underlying mechanisms are not fully understood yet. Previous evidence shows that HFD intake induces the activation of microglia, which is mainly dependent on the increased expression of Iba1 in various regions of the brain in rodents [43,44]. In our study, the expressions of genes related to neuronal development and microglia-neuron interaction in both hippocampus and cortex were decreased in the HFD group compared to those in the ND group. However, the expression of the genes involved in microglia maturation did not change significantly between ND- and HFD-fed mice, implying factors other than HFD may be required to cause substantial damage to the maturation of microglia. The dysregulation of synaptic function and ultrastructure has been reported in neurobehavioral disorders, such as cognitive impairment and AD [45,46]. In the present study, it was found that the expression of genes related to synaptic-plasticity in the hippocampus and cortex was lower in HFD-fed mice than those in ND-fed mice.

The gut-brain axis referring to the bidirectional communication between gut bacteria and the peripheral nervous system, as well as the gut microbial-derived metabolites to pass through BBB to the brain, is critical and important in maintaining homeostasis of the gastrointestinal, central nervous and gut microbial systems of rodents [47,48]. In fact, this axis is associated with many neuropsychiatric diseases of neurodevelopment, neurodegeneration and behavior, suggesting that gut-brain axis occurs throughout the whole lifespan [11]. In this study, we observed that prolonged HFD consumption throughout adulthood altered anxiety- and depression-like behavior, social behavior, and learning and memory. Consistent with previous findings showing that HFD can induce gut microbiota dysbiosis, including structural and functional changes [49,50], this study reported here that the diversity of gut microbiota was decreased and the structures of microbial communities were also significantly altered in HFD-fed mice. It has been reported that *Ruminococcus* of Firmicutes degrades mucus and increases intestinal permeability, while *Bacteroides fragilis* of Bacteriodetes phylum increases tight junction expression and protects mucus from degradation [51,52]. A recent study reported by Leggio’s group showed that Trauma exposure affected mostly the abundance of Firmicutes phylum in the post-traumatic stress disorder susceptible mice. At the Family level, an increased relative abundance of pro-inflammatory-related bacteria, including *Ruminococcaceae* and *Lachnospiraceae*, was detected in the susceptible mice. These species are known to produce toxic metabolites such as ammonium, indole, and p-cresol through the degradation and fermentation of proteins, which are capable of affecting social, anxiety-like and depressive-like behaviors [53]. By contrast, *Akkermansia*, as a beneficial bacterium, has been reported to protect intestinal barrier integrity through the regulation of tight junctions [54]. Here, we observed the increased abundance of Firmicutes and decreased abundance of Bacteriodetes after long-term HFD intake. The related taxa in Firmicutes and Bacteriodetes altered by HFD probably induced behavioral phenotypes related to neuropsychiatric disorders by producing metabolites that could pass through BBB. Moreover, the abundance of *Akkermansia* was significantly higher in ND-fed mice in comparison to those consuming HFD. Decreased gut barrier integrity could trigger a neuroinflammatory response due to the translocation of bacterial LPS into the blood circulation, namely endotoxemia [55]. The thickness of the mucus layer and the expression levels of tight junction proteins were significantly attenuated, while the LPS level and the expression of inflammation in the colon were notably increased after long-term HFD consumption. Collectively, results from this study proved that the dysbiosis of gut microbiota induced by long-term HFD consumption in adult mice could impair intestinal barrier integrity, subsequently inducing neurobehavioral disorders through systemic and neuronal inflammation.

## Data Availability

Not applicable.

## References

[1] Livingston G., Huntley J., Sommerlad A., Ames D., Ballard C., Banerjee S., Brayne C., Burns A., Cohen-Mansfield J., Cooper C. (2020). Dementia prevention, intervention, and care: 2020 report of the Lancet Commission. Lancet.

[2] Dutheil S., Ota K.T., Wohleb E.S., Rasmussen K., Duman R.S. (2016). High-Fat Diet Induced Anxiety and Anhedonia: Impact on Brain Homeostasis and Inflammation. Neuropsychopharmacology.

[3] Zilkha N., Kuperman Y., Kimchi T. (2017). High-fat diet exacerbates cognitive rigidity and social deficiency in the BTBR mouse model of autism. Neuroscience.

[4] Cordner Z.A., Tamashiro K.L. (2015). Effects of high-fat diet exposure on learning & memory. Physiol. Behav..

[5] Kesby J., Kim J.J., Scadeng M., Woods G., Kado D.M., Olefsky J.M., Jeste D.V., Achim C.L., Semenova S. (2015). Spatial Cognition in Adult and Aged Mice Exposed to High-Fat Diet. PLoS ONE.

[6] Zhuang H., Yao X., Li H., Li Q., Yang C., Wang C., Xu D., Xiao Y., Gao Y., Gao J. (2022). Long-term high-fat diet consumption by mice throughout adulthood induces neurobehavioral alterations and hippocampal neuronal remodeling accompanied by augmented microglial lipid accumulation. Brain Behav. Immun..

[7] Bruce-Keller A.J., Salbaum J.M., Luo M., Blanchard E., Taylor C.M., Welsh D.A., Berthoud H.-R. (2015). Obese-type gut microbiota induce neurobehavioral changes in the absence of obesity. Biol. Psychiatry.

[8] Agustí A., García-Pardo M.P., López-Almela I., Campillo I., Maes M., Romani-Pérez M., Sanz Y. (2018). Interplay Between the Gut-Brain Axis, Obesity and Cognitive Function. Front. Neurosci..

[9] Sharon G., Sampson T.R., Geschwind D.H., Mazmanian S.K. (2016). The Central Nervous System and the Gut Microbiome. Cell.

[10] Erny D., Hrabě de Angelis A.L., Jaitin D., Wieghofer P., Staszewski O., David E., Keren-Shaul H., Mahlakoiv T., Jakobshagen K., Buch T. (2015). Host microbiota constantly control maturation and function of microglia in the CNS. Nat. Neurosci..

[11] Morais L.H., Schreiber H.L., Mazmanian S.K. (2021). The gut microbiota-brain axis in behaviour and brain disorders. Nat. Rev. Microbiol..

[12] Fuke N., Nagata N., Suganuma H., Ota T. (2019). Regulation of Gut Microbiota and Metabolic Endotoxemia with Dietary Factors. Nutrients.

[13] Tarassishin L., Suh H.S., Lee S.C. (2014). LPS and IL-1 differentially activate mouse and human astrocytes: Role of CD14. Glia.

[14] Valcarcel-Ares M.N., Tucsek Z., Kiss T., Giles C.B., Tarantini S., Yabluchanskiy A., Balasubramanian P., Gautam T., Galvan V., Ballabh P. (2019). Obesity in Aging Exacerbates Neuroinflammation, Dysregulating Synaptic Function-Related Genes and Altering Eicosanoid Synthesis in the Mouse Hippocampus: Potential Role in Impaired Synaptic Plasticity and Cognitive Decline. J. Gerontol. A Biol. Sci. Med. Sci..

[15] Kraeuter A.K., Guest P.C., Sarnyai Z. (2019). The Open Field Test for Measuring Locomotor Activity and Anxiety-Like Behavior. Methods Mol. Biol..

[16] Kaidanovich-Beilin O., Lipina T., Vukobradovic I., Roder J., Woodgett J.R. (2011). Assessment of social interaction behaviors. J. Vis. Exp..

[17] Deacon R.M. (2006). Assessing nest building in mice. Nat. Protoc..

[18] Dixit P.V., Sahu R., Mishra D.K. (2020). Marble-burying behavior test as a murine model of compulsive-like behavior. J. Pharmacol. Toxicol. Methods.

[19] Lueptow L.M. (2017). Novel Object Recognition Test for the Investigation of Learning and Memory in Mice. J. Vis. Exp..

[20] Othman M.Z., Hassan Z., Che Has A.T. (2022). Morris water maze: A versatile and pertinent tool for assessing spatial learning and memory. Exp. Anim..

[21] Chen S., Zhou Y., Chen Y., Gu J. (2018). fastp: An ultra-fast all-in-one FASTQ preprocessor. Bioinformatics.

[22] Magoč T., Salzberg S.L. (2011). FLASH: Fast length adjustment of short reads to improve genome assemblies. Bioinformatics.

[23] Edgar R.C. (2013). UPARSE: Highly accurate OTU sequences from microbial amplicon reads. Nat. Methods.

[24] Wang Q., Garrity G.M., Tiedje J.M., Cole J.R. (2007). Naive Bayesian classifier for rapid assignment of rRNA sequences into the new bacterial taxonomy. Appl. Environ. Microbiol..

[25] Wen Y.R., Tan P.H., Cheng J.K., Liu Y.C., Ji R.R. (2011). Microglia: A promising target for treating neuropathic and postoperative pain, and morphine tolerance. J. Formos. Med. Assoc..

[26] Malesza I.J., Malesza M., Walkowiak J., Mussin N., Walkowiak D., Aringazina R., Bartkowiak-Wieczorek J., Mądry E. (2021). High-Fat, Western-Style Diet, Systemic Inflammation, and Gut Microbiota: A Narrative Review. Cells.

[27] Paeratakul S., Ferdinand D.P., Champagne C.M., Ryan D.H., Bray G.A. (2003). Fast-food consumption among US adults and children: Dietary and nutrient intake profile. J. Am. Diet. Assoc..

[28] Kopp W. (2019). How Western Diet and Lifestyle Drive the Pandemic of Obesity and Civilization Diseases. Diabetes Metab. Syndr. Obes..

[29] Eskelinen M.H., Ngandu T., Helkala E., Tuomilehto J., Nissinen A., Soininen H., Kivipelto M. (2008). Fat intake at midlife and cognitive impairment later in life: A population-based CAIDE study. Int. J. Geriatr. Psychiatry.

[30] Pasinetti G.M., Eberstein J.A. (2008). Metabolic syndrome and the role of dietary lifestyles in Alzheimer’s disease. J. Neurochem..

[31] Winocur G., Greenwood C.E., Piroli G.G., Grillo C.A., Reznikov L.R., Reagan L.P., McEwen B.S. (2005). Memory impairment in obese Zucker rats: An investigation of cognitive function in an animal model of insulin resistance and obesity. Behav. Neurosci..

[32] Boehme M., van de Wouw M., Bastiaanssen T.F., Olavarría-Ramírez L., Lyons K., Fouhy F., Golubeva A.V., Moloney G.M., Minuto C., Sandhu K.V. (2020). Mid-life microbiota crises: Middle age is associated with pervasive neuroimmune alterations that are reversed by targeting the gut microbiome. Mol. Psychiatry.

[33] Schubert C.R., Fischer M.E., Pinto A.A., Chen Y., Klein B.E., Klein R., Tsai M.Y., Tweed T.S., Cruickshanks K.J. (2019). Brain Aging in Midlife: The Beaver Dam Offspring Study. J. Am. Geriatr. Soc..

[34] Carola V., D’Olimpio F., Brunamonti E., Mangia F., Renzi P. (2002). Evaluation of the elevated plus-maze and open-field tests for the assessment of anxiety-related behaviour in inbred mice. Behav. Brain Res..

[35] Khoo S., Morris T. (2012). Physical activity and obesity research in the Asia-Pacific: A review. Asia Pac. J. Public Health.

[36] Rivera H.M., Christiansen K.J., Sullivan E.L. (2015). The role of maternal obesity in the risk of neuropsychiatric disorders. Front. Neurosci..

[37] Deng W., Ke H., Wang S., Li Z., Li S., Lv P., Li F., Chen Y. (2022). Metformin Alleviates Autistic-Like Behaviors Elicited by High-Fat Diet Consumption and Modulates the Crosstalk Between Serotonin and Gut Microbiota in Mice. Behav. Neurol..

[38] Christian Furlan F., Marriott A., Gill D., Kaleko M. (2020). Maternal treatment with oral intestinal alkaline phosphatase mitigates high fat diet-induced cognitive disorders in offspring mice. Behav. Brain Res..

[39] Bromley-Brits K., Deng Y., Song W. (2011). Morris water maze test for learning and memory deficits in Alzheimer’s disease model mice. J. Vis. Exp..

[40] Liu W., Ge T., Leng Y., Pan Z., Fan J., Yang W., Cui R. (2017). The Role of Neural Plasticity in Depression: From Hippocampus to Prefrontal Cortex. Neural Plast..

[41] MacQueen G., Frodl T. (2011). The hippocampus in major depression: Evidence for the convergence of the bench and bedside in psychiatric research?. Mol. Psychiatry.

[42] Spinelli M., Fusco S., Mainardi M., Scala F., Natale F., Lapenta R., Mattera A., Rinaudo M., Puma D.D.L., Ripoli C. (2017). Brain insulin resistance impairs hippocampal synaptic plasticity and memory by increasing GluA1 palmitoylation through FoxO3a. Nat. Commun..

[43] Reichelt A.C., Lemieux C.A., Princz-Lebel O., Singh A., Bussey T.J., Saksida L.M. (2021). Age-dependent and region-specific alteration of parvalbumin neurons, perineuronal nets and microglia in the mouse prefrontal cortex and hippocampus following obesogenic diet consumption. Sci. Rep..

[44] Spencer S.J., Basri B., Sominsky L., Soch A., Ayala M.T., Reineck P., Gibson B.C., Barrientos R.M. (2019). High-fat diet worsens the impact of aging on microglial function and morphology in a region-specific manner. Neurobiol. Aging.

[45] Head E., Corrada M.M., Kahle-Wrobleski K., Kim R.C., Sarsoza F., Goodus M., Kawas C.H. (2009). Synaptic proteins, neuropathology and cognitive status in the oldest-old. Neurobiol. Aging.

[46] Whitfield D.R., Vallortigara J., Alghamdi A., Howlett D., Hortobágyi T., Johnson M., Attems J., Newhouse S., Ballard C., Thomas A.J. (2014). Assessment of ZnT3 and PSD95 protein levels in Lewy body dementias and Alzheimer’s disease: Association with cognitive impairment. Neurobiol. Aging.

[47] Cryan J.F., O’Riordan K.J., Cowan C.S., Sandhu K.V., Bastiaanssen T.F., Boehme M., Codagnone M.G., Cussotto S., Fulling C., Golubeva A.V. (2019). The Microbiota-Gut-Brain Axis. Physiol. Rev..

[48] Martin C.R., Osadchiy V., Kalani A., Mayer E.A. (2018). The Brain-Gut-Microbiome Axis. Cell Mol. Gastroenterol. Hepatol..

[49] Bibbò S., Ianiro G., Giorgio V., Scaldaferri F., Masucci L., Gasbarrini A., Cammarota G. (2016). The role of diet on gut microbiota composition. Eur. Rev. Med. Pharmacol. Sci..

[50] Wang P., Gao J., Ke W., Wang J., Li D., Liu R., Jia Y., Wang X., Chen X., Chen F. (2020). Resveratrol reduces obesity in high-fat diet-fed mice via modulating the composition and metabolic function of the gut microbiota. Free Radic. Biol. Med..

[51] Hynönen U., Rasinkangas P., Satokari R., Paulin L., de Vos W.M., Pietilä T.E., Kant R., Palva A. (2016). Isolation and whole genome sequencing of a Ruminococcus-like bacterium, associated with irritable bowel syndrome. Anaerobe.

[52] Hsiao E.Y., McBride S.W., Hsien S., Sharon G., Hyde E.R., McCue T., Codelli J.A., Chow J., Reisman S.E., Petrosino J.F. (2013). Microbiota modulate behavioral and physiological abnormalities associated with neurodevelopmental disorders. Cell.

[53] Laudani S., Torrisi S.A., Alboni S., Bastiaanssen T.F., Benatti C., Rivi V., Moloney R.D., Fuochi V., Furneri P.M., Drago F. (2023). Gut microbiota alterations promote traumatic stress susceptibility associated with p-cresol-induced dopaminergic dysfunctions. Brain Behav. Immun..

[54] Chelakkot C., Choi Y., Kim D.K., Park H.T., Ghim J., Kwon Y., Jeon J., Kim M.-S., Jee Y.-K., Gho Y. (2018). Akkermansia muciniphila-derived extracellular vesicles influence gut permeability through the regulation of tight junctions. Exp. Mol. Med..

[55] Podbielska M., Das A., Smith A.W., Chauhan A., Ray S.K., Inoue J., Azuma M., Nozaki K., Hogan E.L., Banik N.L. (2016). Neuron-microglia interaction induced bi-directional cytotoxicity associated with calpain activation. J. Neurochem..

